# The Patterns of Histone Modifications in the Vicinity of Transcription Factor Binding Sites in Human Lymphoblastoid Cell Lines

**DOI:** 10.1371/journal.pone.0060002

**Published:** 2013-03-19

**Authors:** Yumin Nie, Hongde Liu, Xiao Sun

**Affiliations:** State Key Laboratory of Bioelectronics, School of Biological Science & Medical Engineering, Southeast University, Nanjing, China; Georgia Institute of Technology, United States of America

## Abstract

Transcription factor (TF) binding at specific DNA sequences is the fundamental step in transcriptional regulation and is highly dependent on the chromatin structure context, which may be affected by specific histone modifications and variants, known as histone marks. The lack of a global binding map for hundreds of TFs means that previous studies have focused mainly on histone marks at binding sites for several specific TFs. We therefore studied 11 histone marks around computationally-inferred and experimentally-determined TF binding sites (TFBSs), based on 164 and 34 TFs, respectively, in human lymphoblastoid cell lines. For H2A.Z, methylation of H3K4, and acetylation of H3K27 and H3K9, the mark patterns exhibited bimodal distributions and strong pairwise correlations in the 600-bp region around enriched TFBSs, suggesting that these marks mainly coexist within the two nucleosomes proximal to the TF sites. TFs competing with nucleosomes to access DNA at most binding sites, contributes to the bimodal distribution, which is a common feature of histone marks for TF binding. Mark H3K79me2 showed a unimodal distribution on one side of TFBSs and the signals extended up to 4000 bp, indicating a longer-distance pattern. Interestingly, H4K20me1, H3K27me3, H3K36me3 and H3K9me3, which were more diffuse and less enriched surrounding TFBSs, showed unimodal distributions around the enriched TFBSs, suggesting that some TFs may bind to nucleosomal DNA. Besides, asymmetrical distributions of H3K36me3 and H3K9me3 indicated that repressors might establish a repressive chromatin structure in one direction to repress gene expression. In conclusion, this study demonstrated the ranges of histone marks associated with TF binding, and the common features of these marks around the binding sites. These findings have epigenetic implications for future analysis of regulatory elements.

## Introduction

Most eukaryotic genomic DNA is packaged into chromatin structure to achieve high compaction. The basic units of chromatin structure are nucleosomes, consisting of an octamer of four core histones (H2A, H2B, H3 and H4) wrapped in 146 base pairs (bps) of DNA [1], [2]. Nucleosomal histones are subject to specific posttranslational modifications and variants, known as histone marks, which may affect the chromatin structure and thus play crucial roles in regulating gene expression in a cell-type-specific manner [3]–[7]. A comprehensive analysis of 39 different histone methylation and acetylation marks in human CD4+T cells has indicated that most modifications, except H3K27me2, H3K27me3, H3K9me2, H3K9me3 and H4K20me3, are associated with gene activation [8], [9], and specific combinations of chromatin marks are correlated with various genomic regions [5], [10]. For example, H3K4me2, H3K4me3, histone acetylation and H2A.Z are commonly located in active promoters, while H3K36me3 and H3K79me2 are enriched in transcribed regions [5].

Transcription factors (TFs) bind to specific DNA sequences and interact with components of the polymerase complex or with other complexes to initiate transcription in eukaryotes, and this process is highly associated with specific histone variants and modifications [11]–[14]. It has been suggested that enhancers are characterized by H3K4me1 and H3K4me2 [15], [16], while CTCF (CCCTC-binding factor) binding sites are enriched with H2A.Z, H3K9me1 and all three states of H3K4 methylation and may function as barriers separating active and repressive regions of chromatin [8], [17]. However, TF binding is a dynamic process that varies between species, individuals within the same species, and even alleles within the same individual [18], making it difficult to identify binding locations for large numbers of factors in specific cell types. Previous studies have therefore focused mainly on histone marks around binding sites for few specific TFs.

However, the development of experimental technology and computational algorithms has permitted further progress in detecting TF binding along the genome. Recent advances in chromatin immunoprecipitation followed by sequencing (ChIP-seq) have allowed investigators to determine experimentally the genome-wide binding locations for specific TFs in a given cell type [12], [19], [20]. However, ChIP-seq is only applied to one TF in a single experiment, and it would therefore be time-consuming to create a global binding map for all TFs in the human genome by ChIP-seq. Computational methods that integrate genome sequence information and cell-specific experimental data, however, have the advantage of allowing accurate profiles for many factors in a specific sample to be determined [21]–[23], and a genome-wide map of 827,000 TF binding sites (TFBSs) in a human lymphoblastoid cell line has been completed using the CENTIPEDE algorithm [23].

To better understand the relationship between histone modifications and TF binding, we examined the enrichment profiles of 11 available histone marks in different windows at CENTIPEDE and ChIP-seq binding sites, respectively, in a human lymphoblastoid cell line, to provide a basis for the choice of an informative mark window size. The intensities of histone marks at TFBSs are generally represented by the number of tags in a specific window around the site [23], and a site is considered to be enriched with a mark if the tag count exceeds the threshold set for the mark. We subsequently explored the patterns of 11 histone marks and discovered that most marks showed bimodal distributions around binding sites, but differed in the distances between the two local maxima. We then focused on two sets of binding sites classified by the distance of the sites relative to the nearest gene and the functions of the bound TFs, to test whether the marks exhibited distinct patterns around these sites. We finally investigated the pairwise correlations between the histone marks at TFBSs. All the analyses were applied to CENTIPEDE and ChIP-seq sites, respectively.

## Results

### Enrichment of histone marks at binding sites

**Figure 1:**
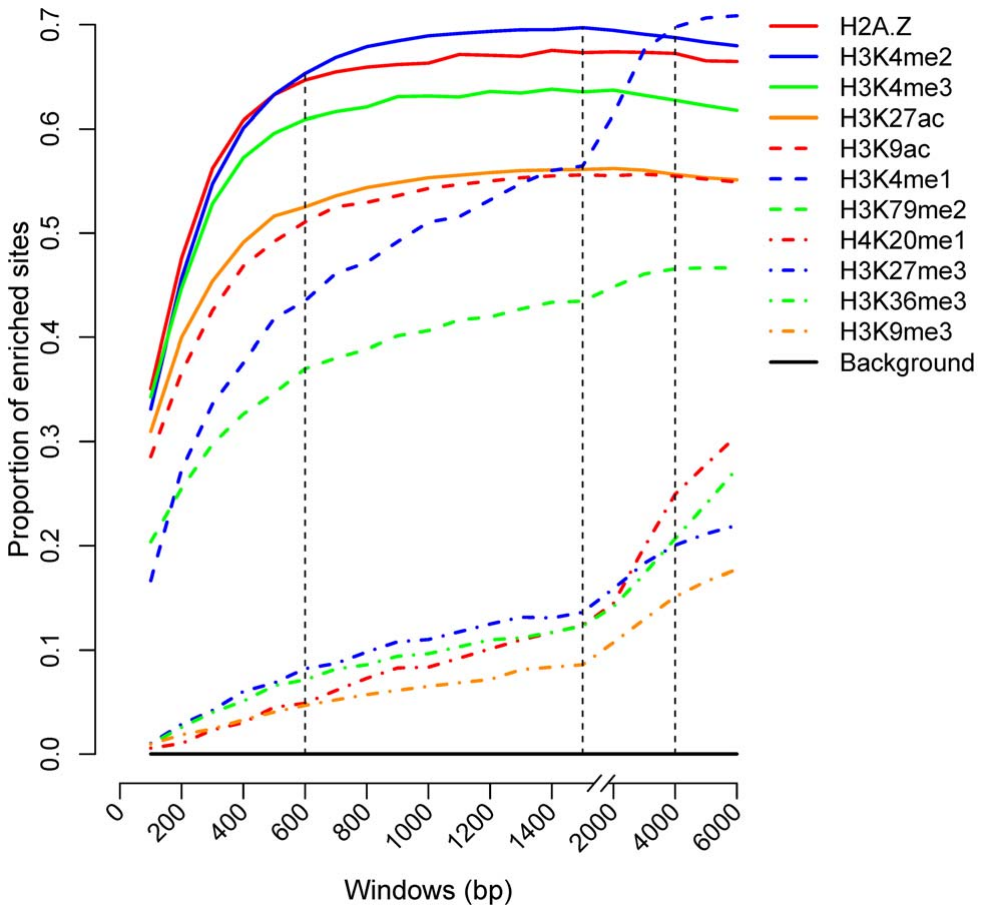
Proportion of CENTIPEDE binding sites enriched with histone marks in different windows. Histone marks showed different tendencies with increasing window size, indicating that the marks were associated with TF binding within different regions around binding sites. Enrichment was determined by comparing the number of tags mapping in the window region surrounding the sites with the threshold set for the mark. Sites with more tags than the threshold were considered as enriched sites.

We examined the relative enrichments of H2A.Z, H3K4me1, H3K4me2, H3K4me3, H3K9ac, H3K27ac, H3K79me2, H4K20me1, H3K27me3, H3K36me3 and H3K9me3 at CENTIPEDE binding sites, as described in the Materials and Methods. The marks were enriched to different degrees in different windows (Figure 1). Previous studies found that some enhancers were marked by histone modification H3K4me1 or H3K4me2 [15], [16], while our results also indicated higher frequencies of H2A.Z, H3K4me3, H3K27ac and H3K9ac at TFBSs. More than 50% of CENTIPEDE sites were enriched with the above marks, except for H3K4me1, at a window size of 600 bp, while 70% of sites were enriched with H3K4me1, with the highest frequency at a window size of 4000 bp.

All the histone marks were more enriched at CENTIPEDE sites compared with the genome as a whole (One-sided binomial test, p<2.2×10^−16^), indicating an association between histone modifications and TF binding in transcription. However, the modified regions associated with binding differed widely between marks (Figure 1). In the cases of H2A.Z, H3K4me2, H3K4me3, H3K27ac and H3K9ac, the percentage of enriched sites remained stable at window sizes greater than 600 bp, indicating that the five marks were associated with binding over a range in the 600-bp region around binding sites. The regions for H3K4me1 and H3K79me2 marks, however, extended to 4000 bp, and were even larger for H4K20me1, H3K27me3, H3K36me3 and H3K9me3. We therefore considered the latter four marks as long-range marks, and the other seven as short-range marks.

The same conclusions were reached by analysing the ChIP-seq binding sites (Figure S1). However, compared with the CENTIPEDE sites, ChIP-seq sites showed a slightly lower enrichment for most of the marks. CENTIPEDE sites tend to be in the proximity of transcription start sites (TSSs) [23], while more ChIP-seq sites are located in the distal regions. We inferred this might account for the higher enrichment of CENTIPEDE sites.

The total number of mapped tags in each sample ranged from 14.5 million (M) to 33.0 M. In order to test whether the different number of tags had a great influence on the definition of the threshold, 10 M tags for each mark were randomly sampled to repeat the enrichment analysis. Both CENTIPEDE and ChIP-seq analyses (Figure S2) indicated a decrease in the proportion of sites enriched with each mark, especially in the cases of H2A.Z, H3K4me1, H3K4me3 and H3K79me2. However, compared with the previous results, each mark showed the similar enrichment tendency with increasing window size, indicating that H2A.Z, H3K4me2, H3K4me3, H3K27ac, H3K9ac, H3K4me1 and H3K79me2 were associated with binding in the 4000-bp region and the regions for H4K20me1, H3K27me3, H3K36me3 and H3K9me3 were longer, which was consistent with the previous definition of short- and long-range marks.

### Patterns of histone marks around binding sites

**Figure 2:**
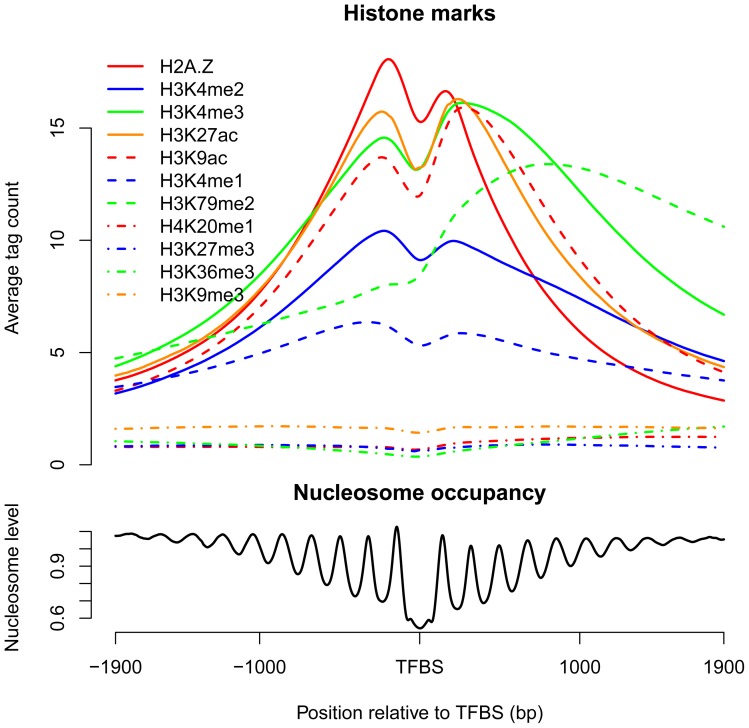
Patterns of histone marks and nucleosome occupancy around all CENTIPEDE binding sites. The short-range marks, except for H3K79me2, showed bimodal distributions around the binding sites, based on the average tag count. The nucleosomes were generally depleted at the binding sites.

To account for the different regions of histone marks, we investigated the mark patterns around the CENTIPEDE (Figure 2) and ChIP-seq sites, respectively (Figure S3). Both sets of results indicated that the six short-range marks H2A.Z, H3K4me2, H3K4me3, H3K27ac, H3K9ac and H3K4me1, which were associated with TF accessibility within 4000 bp, showed bimodal distributions around the binding sites. These short-range marks decreased at the binding sites and decreased with increasing distance in the flanking regions. H3K79me2, which differed from other short-range marks, exhibited a strong peak on one side of TFBSs. Because of their lower enrichments at binding sites, the signals of the long-range marks H4K20me1, H3K27me3, H3K36me3 and H3K9me3 were so weak that the obvious mark patterns were not observed.

We also examined the average nucleosome occupancy around CENTIPEDE sites, and observed nucleosome-free regions (NFRs) at the binding sites (Figure 2), suggesting that the lower mark signals at TFBSs are determined by the nucleosome distribution. Similar NFRs at ChIP-seq sites have been indicated in previous study [24]. The lower signals for histone marks and nucleosome occupancy are necessary for most binding sites, as TFs can compete with nucleosomes to slide or evict them for access to the specific DNA [2], [7], [25]. H2A.Z, H3K4me2, H3K4me3, H3K27ac and H3K9ac showed bimodal signals within 600-bp regions around the binding sites, indicating the existence of two well-positioned nucleosomes flanking the binding sites, which might form a chromatin configuration to facilitate TF binding [7], [25]. In addition, some marks, such as H3K4me3 and H3K4me2, might recruit chromatin modifiers [26] or pioneer factors [16], [27] to enable the access of TFs to DNA, and histone acetylation might regulate DNA accessibility directly by disrupting electrostatic interactions between histones and DNA to induce local chromatin changes [26]. However, the patterns of histone modifications around TFBSs might simply be the consequence of dynamic nucleosome remodeling and transcription [28].

The four long-range marks showed a lower enrichment at TFBSs. To further explore the distribution patterns of long-range marks, both CENTIPEDE and ChIP-seq sites were classified as enriched or non-enriched, based on the tag count of a mark in the 600-bp window (most informative for most marks) around the sites. If the tag count exceeded the threshold determined by the mark distribution in the genome and the local tag count in the control sample, the sites were considered to be enriched. We then investigated the mark signals around enriched and non-enriched sites and found that all four long-range marks showed unimodal distributions around enriched sites (Figure S4). The higher signals surrounding enriched TFBSs indicated the presence of a central nucleosome or nucleosomes modified with the long-range marks. It has been suggested that several pioneer factors have the ability to access target sequences on nucleosomes [27], and the TF NF-κB p50 can also bind to nucleosomal DNA without perturbing the overall structure of the nucleosome [29]. It should be noted that TFs may bind to DNA on nucleosomes containing short-range marks. For example, H3K4me1-marked nucleosomes have been suggested to locate at binding sites of the nuclear hormone receptor HNF4A in mouse liver [30].

**Figure 3:**
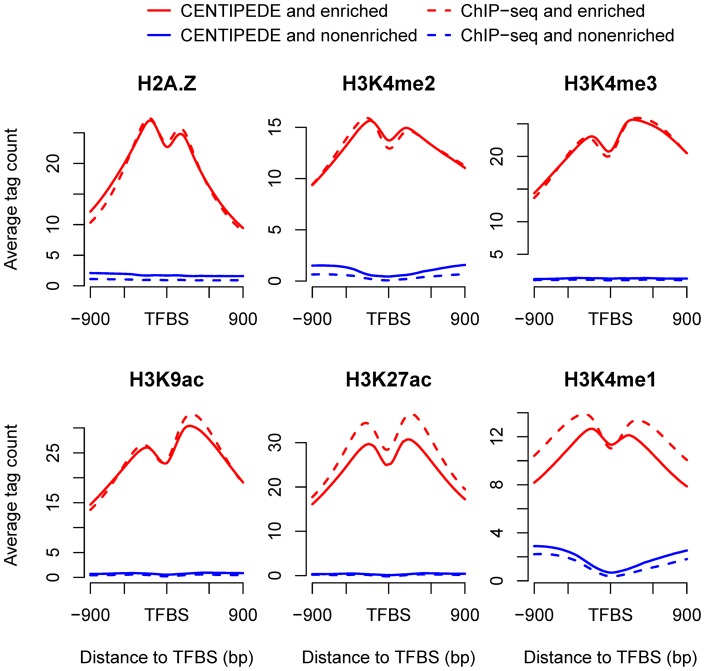
Patterns of six short-range marks around enriched and non-enriched sites by CENTIPEDE and ChIP-seq. The distance between the two local maxima for H3K4me1 was less than 600 bp. Higher H3K4me1 signal intensity around non-enriched sites showed a distinct pattern from other histone marks. Whether a mark was enriched at TFBSs was determined by the tag count in the 600-bp window and the threshold set. Enriched sites were defined as sites with higher tag counts than the threshold.

The same analyses for the short-range marks demonstrated that all the short-range marks except H3K79me2 showed bimodal distributions around enriched sites (Figure 3), as described in Figure 2. For H2A.Z, H3K4me1, H3K4me2, H3K4me3, H3K27ac and H3K9ac, the two local maxima were located within the 600-bp region around enriched sites and the signals outside the region decreased drastically. It was interesting to note that the signal for H3K4me1 was low at the non-enriched sites and significantly higher in the flanking regions, which differed from other marks that were at a low level both at and around the non-enriched sites. Previous analyses indicated that the average H3K4me1 mark signal for all sites showed a 4000-bp region associated with binding, then decreased slowly in the flanking regions; however, a drastic decline was seen for the enriched sites. The longer region for the H3K4me1 mark depended on the signal distribution around the non-enriched sites.

**Figure 4:**
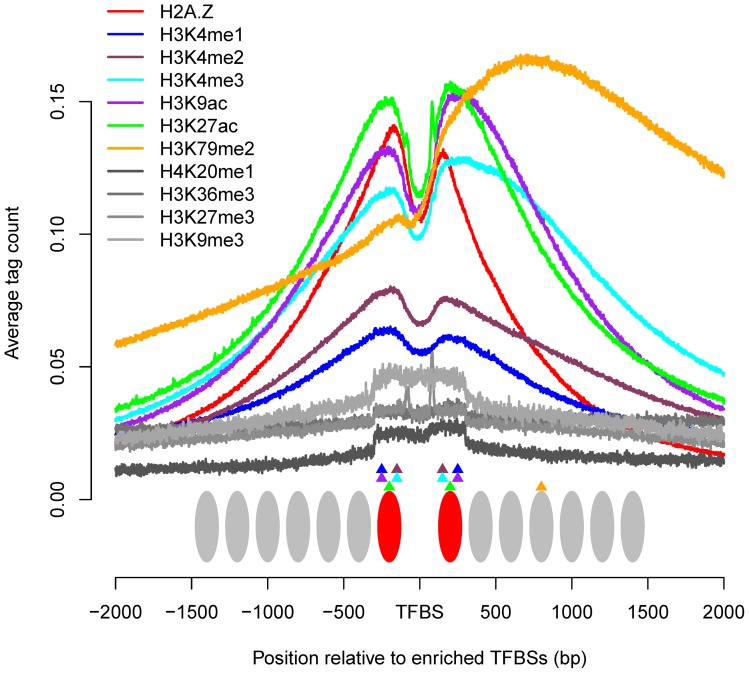
Schematic diagram illustrating the chromatin patterns around enriched TFBSs. H2A.Z, H3K4me1, H3K4me2, H3K4me3, H3K9ac and H3K27ac were located mainly within the two nucleosomes proximal to the enriched sites, while the one local maxima for H3K79me2 were located within the forth nucleosome on one side of TFBSs. The mark signals were not smoothed. The red ellipses represent H2A.Z-containing nucleosomes; the gray ellipses represent typical nucleosomes; the triangles represent different histone modifications.

The histone marks displayed distinct patterns around enriched binding sites, and the regions of marks associated with TF binding differed widely (Figure 4). For H2A.Z, H3K4me1, H3K4me2, H3K4me3, H3K9ac and H3K27ac, the signals were mainly confined within the two nucleosomes proximal to TFBSs. For mark H3K79me2, the local maxima was expanded to ∼720 bp, corresponding to the forth nucleosome on the right side of TFBSs, and the signal showed a slow decline in the flanking regions, leading to a longer region correlating with TF accessibility. The signals in the flanking regions were more diffuse for H4K20me1, H3K36me3, H3K27me3 and H3K9me3, indicating a much broader region associated with TF binding. Although NFRs were necessary for most TFBSs, the higher signals for the four long-range marks at enriched binding sites suggested that some TFs might bind to nucleosomal DNA.

### Histone marks around proximal and distal binding sites

**Figure 5:**
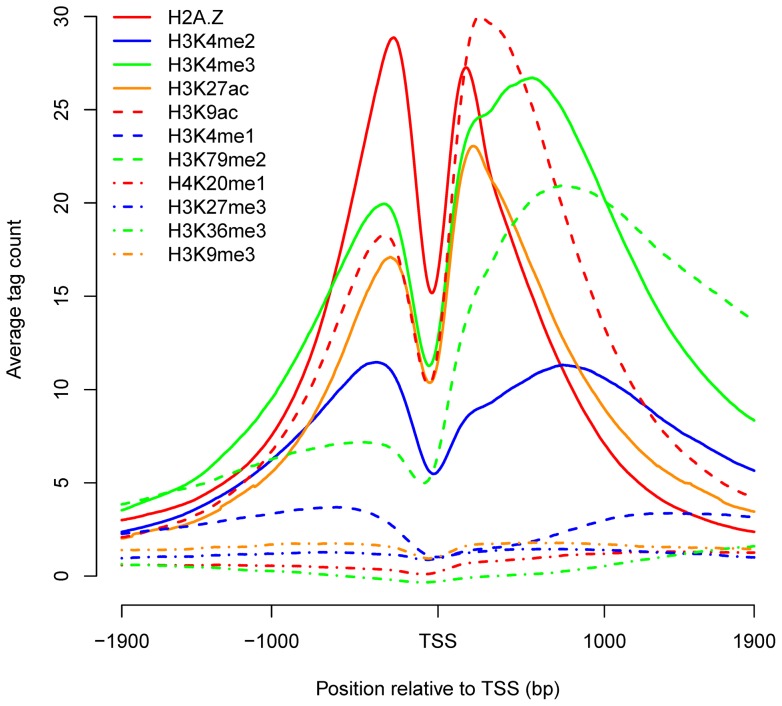
Patterns of histone marks near transcription start sites (TSSs). Three short-range marks H2A.Z, H3K27ac and H3K9ac also displayed bimodal distributions around TSSs, and the signals for most marks were asymmetrical.

**Figure 6:**
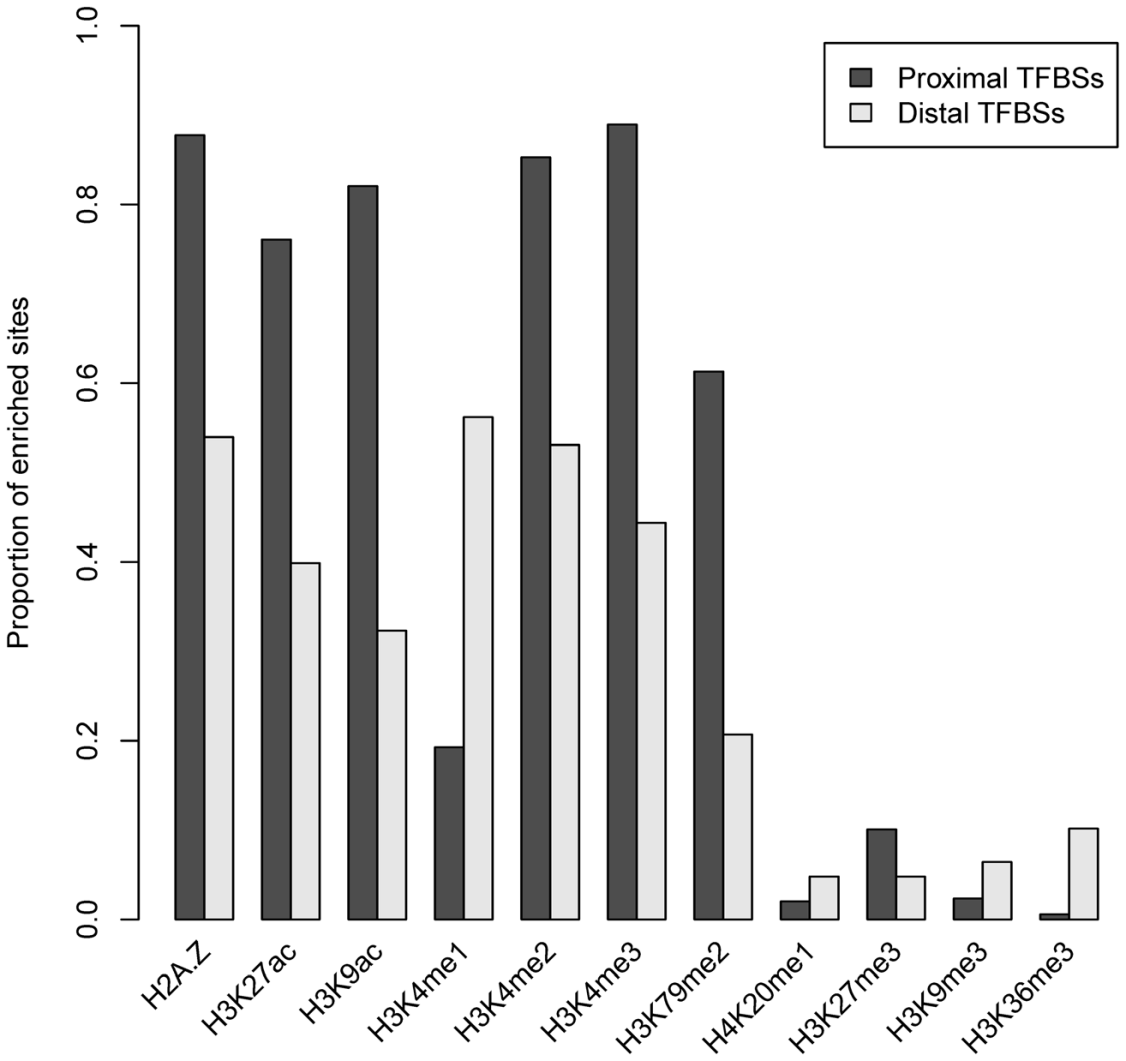
Enrichment of the CENTIPEDE proximal and distal sites in a 600-bp window. The short-range marks, except for H3K4me1, showed significantly higher enrichment at proximal sites.

**Figure 7:**
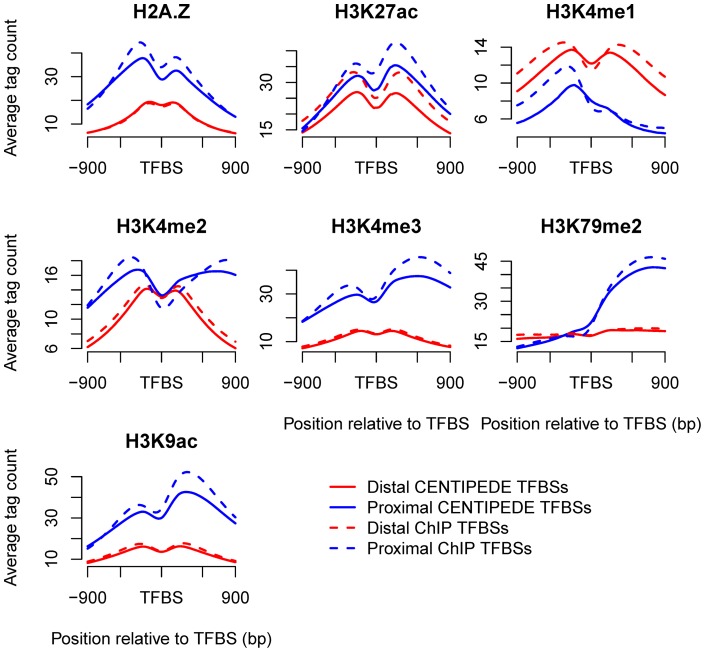
Patterns of seven short-range marks around enriched proximal and distal sites. H2A.Z, H3K4me2, H3K4me3, H3K27ac and H3K9ac marks exhibited significant bimodal distributions around enriched proximal sites, while H3K4me1 showed a bimodal distribution at enriched distal sites.

NFRs are also essential for TSSs, to allow the binding of the transcription machinery [2], [7]. It has been suggested that the histone variant H2A.Z is enriched in the two nucleosomes surrounding TSSs [31], which is consistent with the patterns around TFBSs. Our analyses indicated that marks H3K9ac and H3K27ac surrounding TSSs also showed the similar patterns with that around TFBSs (Figure 5). To examine whether the bimodal distributions around TFBSs were limited in the promoter regions, we distinguished proximal and distal binding sites. The binding sites in the core or proximal promoter are typically located within 1 kb, while distal sites may be situated up to several hundred kb pairs from the core promoter [14]. Here, we defined proximal and distal sites as those located within 1 kb and beyond 10 kb of the nearest TSS, respectively. We investigated the proportions of enriched proximal and distal sites (Figure 6 and Figure S5) and found that the short-range marks, except for H3K4me1, were significantly more enriched at proximal sites (Wilcoxon signed-rank test, p<2.2×10^−16^). Correspondingly, the patterns of H2A.Z, H3K4me2, H3K4me3, H3K27ac and H3K9ac marks (Figure 7) indicated higher average signal intensities and significant bimodal distributions around enriched proximal sites. H3K4me1 showed a smaller proportion of enriched proximal sites and a bimodal distribution at enriched distal sites, indicating that H3K4me1 was located preferentially distal to the core promoter; indeed, it has been suggested that distal enhancers are marked by H3K4me1 [15]. The short-range marks except H3K79me2 also showed bimodal distributions around the distal sites, though some patterns were less noticeable than that around the proximal sites, suggesting the common features of TF binding to DNA. Besides, the mark patterns around distal sites were more symmetrical, indicating that TF binding to the distal sites was independent of the orientation. The three types of distal regulatory elements, including enhancers, silencers and insulators, have been suggested to function in orientation-independent manners [14].

TFs competing with nucleosomes to access DNA may contribute to the bimodal distributions of histone marks around TFBSs. However, the bimodal patterns may be because TFs bind directly to sites in the NFRs flanked by modified nucleosomes, especially for TFBSs nearby TSSs. Assembly of the transcription initiation complex results in NFRs at TSSs in many organisms [7] and several short-range marks have been suggested to display similar bimodal distributions near TSSs [8], [31]. The percentage of proximal sites in CENTIPEDE and ChIP-seq dataset was 31.4% and 20.1%, respectively, among which 27.9% and 38.2% of sites, respectively, were located within 100 bp relative to TSSs, indicating that many proximal sites are indeed located within NFRs flanked by modified nucleosomes and thus mark patterns around proximal sites partially reflect that near TSSs. In addition, all the short-range marks, except for H3K4me1, showed higher average signals around proximal sites (Figure 7). H3K79me2 has been suggested to be located towards the 5′ end of the gene [32], [33], and thus exhibited a strong peak on the right side of TFBSs (Figure 2 and Figure S3). The higher signals around proximal sites can also explain why CENTIPEDE sites (Figure 1), which tend to be in the proximity of TSSs, showed greater enrichments for most of the marks than ChIP-seq sites (Figure S1).

### Histone marks around activator and repressor binding sites

**Figure 8:**
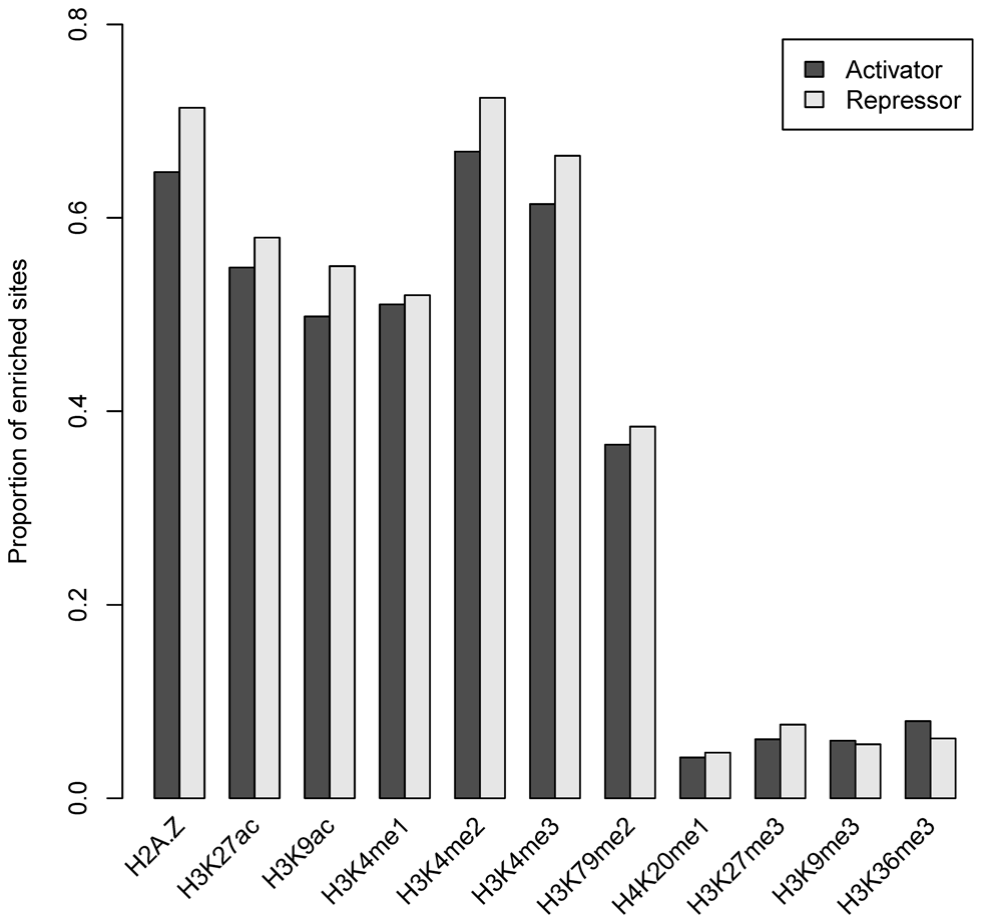
Enrichment of CENTIPEDE activator and repressor sites in a 600-bp window. Most marks showed a slight difference in enrichment percentage between activator and repressor sites.

Previous studies have suggested that some marks, such as H3K27me3 and H3K9me3, are correlated with gene repression, while other marks are correlated with gene activation [9]. Activators and repressors positively and negatively regulate the transcription respectively. We detected similar levels of enrichment of different histone marks at the activator and repressor binding sites according to analyses of both CENTIPEDE (Figure 8) and ChIP-seq sites (Figure S6).

**Figure 9:**
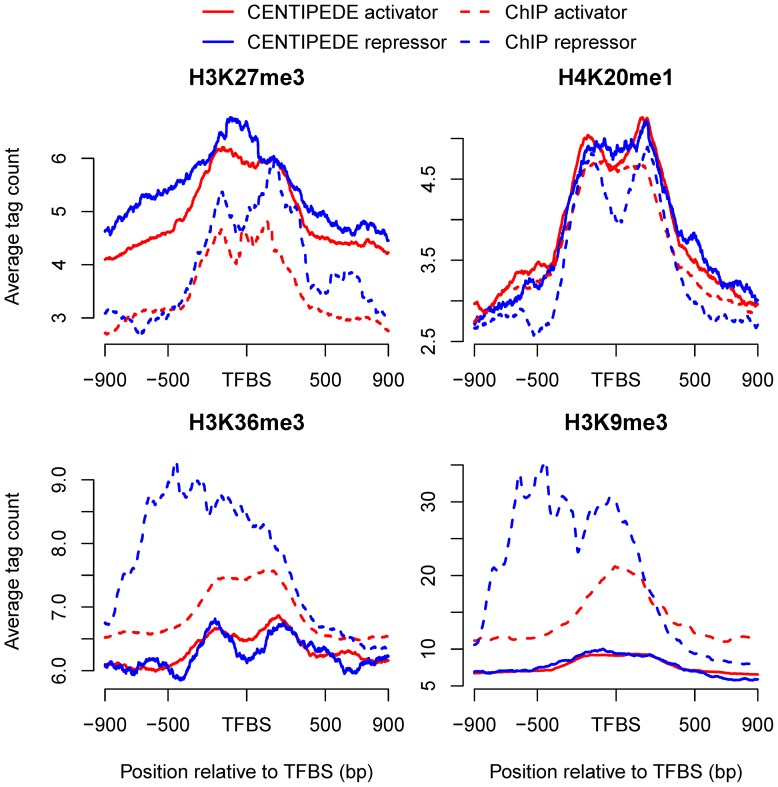
Distribution patterns of long-range marks around enriched activator and repressor binding sites in the distal regions. The patterns of H3K36me3 and H3K9me3 differed in CENTIPEDE and ChIP-seq analyses, and also between the enriched activator and repressor sites.

Further investigations of the signal intensity distributions revealed similar patterns for the seven short-range marks around enriched activator and repressor binding sites in the distal regions, but not for long-range marks. Here we only considered the distal activator and repressor sites to avoid the influence of TSSs. Each of the six short-range marks H2A.Z, H3K4me1, H3K4me2, H3K4me3, H3K27ac and H3K9ac showed similar bimodal distributions around enriched activator and repressor sites according to both CENTIPEDE and ChIP-seq analyses (Figure S7). However, the signal intensities of the two long-range marks H3K36me3 and H3K9me3 differed between CENTIPEDE and ChIP-seq analyses, and also between the enriched activator and repressor binding sites (Figure 9). CENTIPEDE sites were identified based on DNase-seq data reflecting chromatin accessibility [34], and were more likely to be associated with open chromatin [23], CENTIPEDE analysis thus showed similar mark patterns for activator and repressor binding sites. However, the two marks differed widely around activator and repressor binding sites in the ChIP-seq analysis, suggesting different regulatory mechanisms of activators and repressors. In some cases, repressors may compete for the same site with an activator [35]. Alternatively, repressors may establish a repressive chromatin structure to prevent the activator accessing a promoter [14], [36]. H3K36me3 and H3K9me3 showed higher signal intensities on one side of the repressor sites in ChIP-seq analysis, suggesting that some repressors might establish a repressive chromatin structure in one direction. It should be noted that the average signal intensities for long-range marks were much weaker than those for short-range marks, indicating a finite function of long-range marks for TF binding.

### Correlations of histone marks at TFBSs

**Figure 10:**
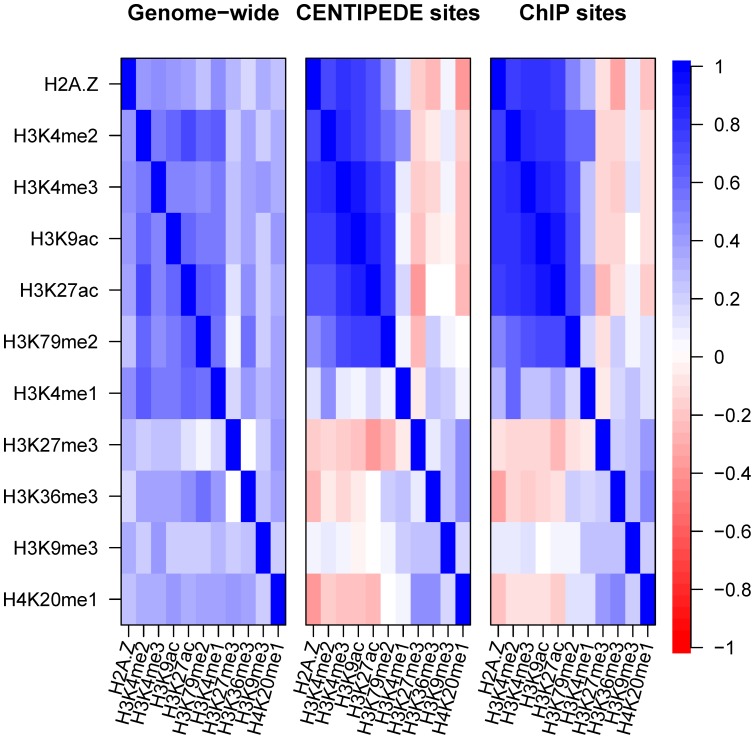
Pairwise correlations of histone marks in the genome and at TFBSs. The five short-range marks H2A.Z, H3K27ac, H3K9ac, H3K4me2 and H3K4me3 showed strong pairwise correlations.

Different histone marks can cross-talk [26], and thus exhibit combinatorial patterns in the genome. It has been suggested that a common module consisting of 17 modifications exists in promoter regions [9]. We examined the modification module at TFBSs by pairwise Spearman correlation analysis of the histone marks, using a 600-bp window. Analyses of both CENTIPEDE and ChIP sites (Figure 10) indicated that the five short-range marks H2A.Z, H3K27ac, H3K9ac, H3K4me2 and H3K4me3 showed strong pairwise correlations (r>0.65; p<2.2×10^−16^), consistent with the notion that these marks coexist in the 600-bp region around the binding sites. H3K79me2 was strongly correlated with H3K27ac, H3K9ac and H3K4me3, but less strongly correlated with H2A.Z and H3K4me2. H3K4me1 and four long-range marks showed weaker correlations with each other and with other marks. The same conclusion was obtained for correlation analyses of activator and repressor binding sites, with little difference between the two types of binding sites (Figure S8). However, the analyses of distal and proximal sites indicated that both H3K4me1 and H3K4me2 were strongly correlated with H2A.Z, H3K27ac, H3K4me3 and H3K9ac at distal, but not proximal sites (Figure S9). The results of previous studies have suggested that H3K4me1 also coexists in the 600-bp region, and is mainly enriched at distal sites, H3K4me1 is thus highly correlated with H2A.Z, H3K27ac, H3K4me2, H3K4me3 and H3K9ac at distal sites.

## Discussion

TF binding at specific DNA sequences is the initial and crucial step of transcription in eukaryotes. Most genomic DNA in eukaryotes is packaged into nucleosomes, and DNA accessibility is thus strongly associated with chromatin structure [2], [7], [11], [25]. Sites in NFRs are easier to access, while the accessibility of sites within nucleosomes depends on the nucleosome dynamics, which are affected by histone variants and modifications, referred to as histone marks [7], [25]. We investigated the patterns of these histone marks around TFBSs, with implications for analyses of regulatory elements.

The signals of histone marks are usually diffuse in broad domains of varying widths, spanning lengths ranging from several nucleosomes to large domains encompassing multiple genes [8], [9], [37], [38]. The number of tags in a given window is generally counted to measure the mark signal. A recent study considered that the tag count in a 400-bp window was the most informative [23], though 200-bp [10], [39] and 1000-bp [40] windows also have been applied to analyses of histone modification data. However, our results detected widely different signal patterns of histone marks around TFBSs, and the choice of window size depended on the specific mark. Each mark of H2A.Z, H3K4me1, H3K4me2, H3K4me3, H3K27ac and H3K9ac showed a bimodal distribution in the proximity of TFBSs, and a drastic decrease in the flanking regions, thus a 600-bp window, corresponding to two nucleosome widths around the binding sites, was sufficient to represent these six marks. H3K79me2 exhibited a unimodal distribution on one side of TFBSs, a 4000–bp window was necessary because of a longer distance between the local maxima and TFBSs, and a gradual decline in the flanking regions. The informative windows for H4K20me1, H3K27me3, H3K36me3 and H3K9me3 have not been determined, because they are associated with TF binding over an exceedingly broad domain. However, median widths for the H3K27me3, H3K9me3 and H3K36me3 domains in human embryonic stem cells of 8.6, 6.9 and 15.7 kb, respectively, have been suggested, increasing to 16.4, 11.4 and 16.9 kb, respectively, in fetal lung fibroblasts [38]. H4K20me1, H3K27me3 and H3K9me3 are considered to be silencing modifications that help to keep most of the genome relatively inaccessible to DNA-binding proteins [26]. Although H3K36me3 is commonly associated with actively transcribed regions, it has also been implicated in transcriptional repression [41]. The large domains for these four marks are mainly determined by their propagation in the heterochromatin or euchromatin [26], [41].

Different regions of histone marks associated with TF binding can be partially explained by the genomic locations where histone marks are known to occur. H2A.Z, H3K4me2, H3K4me3, H3K9ac and H3K27ac have been suggested to peak near TSSs [8], [31] where many activators commonly bind [14], they are thus short-range marks close to TFBSs. Both H3K79me2 and H3K36me3 are associated with the gene body, however, H3K79me2 occurs on nucleosomes closer to TSSs and H3K36me3 is located towards the 3′ end of the gene [32], [33]. That is why H3K79me2 and H3K36me3 are defined as short- and long-range mark, respectively. H4K20me1, H3K27me3 and H3K9me3 are considered to be representative of heterochromatin [5], [26] which is distant from active genes and TFBSs, and hence are long-range marks.

Although previous studies have identified some histone modification patterns at specific enhancers [15], [16], our findings expand the current knowledge of histone modifications at TFBSs. First, the six short-range histone marks examined (H2A.Z, H3K4me1, H3K4me2, H3K4me3, H3K27ac and H3K9ac) displayed bimodal distributions around TFBSs, suggesting the existence of common mark feature that enables TFs to access DNA. Eviction or sliding of nucleosomes at most binding sites contributes to the bimodal distribution. The patterns of these marks were asymmetrical at the proximal sites, because of the interactions between the transcription machinery and other proteins near TSSs, while these marks showed more symmetrical distributions at the distal sites, indicating that TFs bind to DNA in an orientation-independent manner in the distal regions. Second, the four long-range marks (H4K20me1, H3K27me3, H3K36me3 and H3K9me3) were less enriched than the short-range marks at binding sites, and showed unimodal distributions around the enriched sites, suggesting that some TFs may bind to nucleosomal DNA. Third, five short-range marks (H2A.Z, H3K4me2, H3K4me3, H3K27ac and H3K9ac) showed significant bimodal distributions around the proximal sites, which were consistent with the patterns surrounding TSSs. Effects of the transcriptional machinery near TSSs contribute to the greater enrichments and distributions of these marks around the proximal sites. Although H3K4me1, H2A.Z and H3K4me2 showed similar enrichments at distal sites, fewer proximal sites were enriched with H3K4me1, indicating that H3K4me1 tends to be located in the distal regions. All the short-range marks, except for H3K79me2, exhibited bimodal distributions and strong pairwise correlations in the 600-bp region around the distal sites, suggesting that these marks mainly coexist within the two nucleosomes surrounding TFBSs in the distal regions. Fourth, all the short-range marks showed similar distributions around activator and repressor sites, indicating that both activators and repressors require open chromatin to access the specific DNA. However, the patterns of H3K36me3 and H3K9me3 marks differed widely, implying other regulatory mechanisms for repressors. Repressor binding may recruit other proteins and establish a repressive chromatin structure to repress gene expression. For example, in a corepressor molecule CtBP wild-type background, binding of the multifunctional TF YY1 to response elements can recruit Polycomb group proteins that cause histone deacetylation and methylation of H3K9 and H3K27 [36]. Our analyses suggest that repressor-mediated methylation may occur in one direction.

As the key components of chromatin packaging, histone modifications have been implicated in various important biological functions [5]. However, whether the histone modification represents the original element remains a subject for debate in epigenetic research. Combinations of histone modifications, also known as histone codes, have always been considered to bring about distinct downstream events [42], either by influencing the overall chromatin structure directly or by regulating the binding of chromatin molecules [26], while histone modifications and variants may be the consequences of dynamic processes driven primarily by transcription and nucleosome remodeling [28]. Regardless of whether these histone marks are causative factors in directing regulatory processes, or simply maintain the active or silent state of chromatin, identification of histone mark patterns in the regulatory regions may help us to comprehend TF binding processes and identify new functional elements.

Diverse histone marks act cooperatively to affect chromosome packaging and control eukaryotic gene regulation [26]. A common modification module in promoter regions has already been suggested [9], and the results of the current study also indicated strong correlations between several marks. For example, the Spearman correlation coefficient for H3K4me3 and H3K9ac at CENTIPEDE and ChIP-seq sites were 0.90 and 0.88, respectively (p<2.2×10^−16^). TFs can also interact with other TFs, chromatin modifiers and cofactors through protein-protein interactions, and thus affect transcription in combinatorial ways [11], [43]. Future studies to analyse the combinatorial patterns of histone marks around TFBSs and TF-TF interactions are planned.

## Conclusions

We investigated the distribution patterns of 11 histone marks around TFBSs and found wide differences between marks. H2A.Z, H3K4me1, H3K4me2, H3K4me3, H3K27ac and H3K9ac exhibited significant bimodal distributions in the proximity of binding sites, suggesting the existence of common mark features enabling TFs to bind with DNA. These six marks coexist mainly within the two nucleosomes proximal to binding sites. H3K79me2 showed a unimodal distribution on one side of TFBSs and the local maxima was located ∼720 bp relative to TFBSs. H4K20me1, H3K27me3, H3K36me3 and H3K9me3 were more diffuse and less enriched. The latter two marks showed higher signals on one side of enriched sites in the distal regions, indicating the existence of repressors with specific regulatory mechanisms. These results provide insight into the correlations between histone marks and TFBSs, and provide useful epigenetic information for mapping regulatory elements in the genome.

## Materials and Methods

### Data source

ChIP-seq data generated by the Broad/MGH ENCODE group [6], corresponding to 10 histone modifications, one histone variant H2A.Z and input control dataset in the GM12878 cell line, were downloaded from the UCSC ftp server (http://genome.ucsc.edu). Downloaded files display tags aligned to the human reference genome hg19 in BAM format and we therefore converted these to BED format using BEDTools [44]. We then combined the tags for multiple experimental replicates of the same mark and filtered out tags with a quality score <10 [23]. We finally applied a shift of 100 bases to the genomic coordinates of all tags in the 5′ to 3′ direction, as described previously [10]. The nucleosome occupancy data in the GM12878 cell line, generated by the Snyder laboratory, was also downloaded from the UCSC ftp server (http://genome.ucsc.edu).

The hg19 RefSeq gene annotations and the RNA-seq expression data for the GM12878 cell line were obtained from the UCSC website browser (http://genome.ucsc.edu). Non-protein-coding transcripts were excluded. For alternatively spliced transcripts encoding the same protein, only the transcript with the highest expression value was used and transcript expression was quantified in reads per kilobase of exon per million mapped sequence reads [45]. Finally 18,937 TSSs of RefSeq genes were used to investigate the mark patterns around TSSs and define the distance between binding sites and the nearest TSS.

ChIP-seq peak files generated by Myers lab [46], displaying binding regions of 34 TFs along the whole genome in the GM12878 cell line, were downloaded from the UCSC website browser (http://genome.ucsc.edu). The center location of each peak region was considered as the real binding site.

A total of 775,270 binding sites for 164 TFs estimated with CENTIPEDE in the GM12878 cell line, were downloaded from http://centipede.uchicago.edu/. The initial data downloaded were mapped to the human reference genome hg18, and the binding locations were therefore converted from hg18 to hg19 using liftOver, provided by UCSC, and binding sites located on the Y chromosome and unplaced sequences on reference chromosomes were also removed.

### Enrichment of a histone mark

Histone mark intensities at TFBSs were represented by the number of tags in a specific window around the sites, as described previously [23]. To determine whether a histone mark was enriched at a binding location, we identified a threshold based on the distribution of the mark tag count in the genome and the local tag count in the control sample; if the mark tag count in the window around a binding site was greater than the threshold, the mark was considered to be enriched at that site.

The threshold for each mark was calculated as follows: for a specific window size centered at a site, the number of tags in a window X was hypothesized to follow a Poisson distribution parameterized by a dynamic parameter, 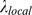, defined as [47]:
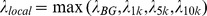
where 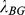 was the expected number of tags mapped to the window by random chance [39], [48] and 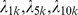 were calculated based on the control tags within the 1 kb, 5 kb and 10 kb centered at the regions of interest [47]. Instead of using a uniform 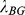 estimated from the whole genome, a dynamic 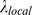 was estimated to capture the local fluctuations and biases in ChIP-seq data. The threshold, t, for each mark at a site was then defined as the smallest integer t such that P(X>t) <10^−4^ [10].

### Distribution of histone marks around TFBSs

For a group of CENTIPEDE or ChIP-seq sites, the mark signals were averaged over all sites after a subtraction of the tag count in the input control data at each position. Except in certain cases, all the mark profiles represented by the average tag count were smoothed with a window of 200 bp when considering the patterns of histone marks.

Promoter upstream to a TSS contains multiple binding sites for activators [14]. Many histone marks have been suggested to be located in specific regions relative to TSSs. For example, H2A.Z and H3K4me2 are enriched upstream of TSSs, while H3K36me3 and H3K79me2 are enriched downstream of TSSs [5], [8], [9]. Asymmetrical distributions of these marks around TSSs are correlated with the direction of transcription. If we overlook the orientation of TFBSs relative to TSSs on the positive and negative strands, and average together many TFBSs that lie upstream of genes on both strands, artificially symmetrical distributions would be obtained around TFBSs. Therefore we assigned each CENTIPEDE and ChIP-seq TFBS to the nearest gene based on its distance to the TSS and reversed the shape profile of binding sites for genes on the negative strand before averaging, to avoid a misleading aggregate.

### Identification of activators and repressors

Activators are proteins that positively regulate transcription or translation, while repressors are proteins that interfere with transcription or repress translation. We queried the UniProt databases [49], a comprehensive resource for protein sequence data and annotation data, to determine if each of the 164 CENTIPEDE TFs and 34 ChIP-seq TFs was an activator or repressor. We then removed some multifunctional TFs that were annotated as both activator and repressor, prior to further analyses. We finally identified 36 activators and nine repressors for CENTIPEDE TFs, and 11 activators and five repressors for ChIP-seq TFs.

### Pairwise correlations of histone marks in the genome

We divided the human genome into non-overlapping 600-bp intervals (the informative window for most histone marks) and counted the adjusted mark tags assigned to each interval, based on the location of the 5′ end of the tag [9], [10]. Finally, the Spearman coefficient for each pair of histone marks was calculated and displayed using R packages (http://www.r-project.org/).

## Supporting Information

Figure S1 **Proportion of ChIP-seq binding sites enriched with histone marks in different windows.**
(TIF)

Figure S2 **Proportion of enriched CENTIPEDE (A) and ChIP-seq (B) sites when a sampling approach was used.**
(TIF)

Figure S3 **Patterns of 11 histone marks around ChIP-seq binding sites.**
(TIF)

Figure S4 **Patterns of long-range marks around CENTIPEDE and ChIP-seq binding sites.**
(TIF)

Figure S5 **Proportion of enriched proximal and distal sites by ChIP-seq in a 600-bp window.**
(TIF)

Figure S6 **Proportion of enriched activator and repressor sites by ChIP-seq in a 600-bp window.**
(TIF)

Figure S7 **Patterns of seven short-range marks around enriched activator and repressor sites in the distal regions inferred by CENTIPEDE and ChIP-seq.**
(TIF)

Figure S8 **Correlations of histone marks at activator and repressor binding sites.**
(TIF)

Figure S9 **Correlations of histone marks at distal and proximal binding sites.**
(TIF)

## References

[1] Luger K, Mader AW, Richmond RK, Sargent DF, Richmond TJ (1997) Crystal structure of the nucleosome core particle at 2.8 A resolution. Nature 389: 251–260. 9305837 10.1038/38444

[2] Bai L, Morozov AV (2010) Gene regulation by nucleosome positioning. Trends Genet 26: 476–483. 20832136 10.1016/j.tig.2010.08.003

[3] Pan Y, Tsai CJ, Ma B, Nussinov R (2010) Mechanisms of transcription factor selectivity. Trends Genet 26: 75–83. 20074831 10.1016/j.tig.2009.12.003 PMC7316385

[4] Huda A, Bowen NJ, Conley AB, Jordan IK (2011) Epigenetic regulation of transposable element derived human gene promoters. Gene 475: 39–48. 21215797 10.1016/j.gene.2010.12.010

[5] Zhou VW, Goren A, Bernstein BE (2011) Charting histone modifications and the functional organization of mammalian genomes. Nat Rev Genet 12: 7–18. 21116306 10.1038/nrg2905

[6] Ernst J, Kheradpour P, Mikkelsen TS, Shoresh N, Ward LD, et al (2011) Mapping and analysis of chromatin state dynamics in nine human cell types. Nature 473: 43–49. 21441907 10.1038/nature09906 PMC3088773

[7] Jiang C, Pugh BF (2009) Nucleosome positioning and gene regulation: advances through genomics. Nat Rev Genet 10: 161–172. 19204718 10.1038/nrg2522 PMC4860946

[8] Barski A, Cuddapah S, Cui K, Roh TY, Schones DE, et al (2007) High-resolution profiling of histone methylations in the human genome. Cell 129: 823–837. 17512414 10.1016/j.cell.2007.05.009

[9] Wang Z, Zang C, Rosenfeld JA, Schones DE, Barski A, et al (2008) Combinatorial patterns of histone acetylations and methylations in the human genome. Nat Genet 40: 897–903. 18552846 10.1038/ng.154 PMC2769248

[10] Ernst J, Kellis M (2010) Discovery and characterization of chromatin states for systematic annotation of the human genome. Nat Biotechnol 28: 817–825. 20657582 10.1038/nbt.1662 PMC2919626

[11] Field Y, Sharon E, Segal E (2011) How transcription factors identify regulatory sites in genomic sequence. In: Hughes TR, editor. Handbook of transcription factors. pp. 193–204. 10.1007/978-90-481-9069-0_9 21557084

[12] Farnham PJ (2009) Insights from genomic profiling of transcription factors. Nat Rev Genet 10: 605–616. 19668247 10.1038/nrg2636 PMC2846386

[13] Wasserman WW, Sandelin A (2004) Applied bioinformatics for the identification of regulatory elements. Nat Rev Genet 5: 276–287. 15131651 10.1038/nrg1315

[14] Maston GA, Evans SK, Green MR (2006) Transcriptional regulatory elements in the human genome. Annu Rev Genomics Hum Genet 7: 29–59. 16719718 10.1146/annurev.genom.7.080505.115623

[15] Heintzman ND, Stuart RK, Hon G, Fu Y, Ching CW, et al (2007) Distinct and predictive chromatin signatures of transcriptional promoters and enhancers in the human genome. Nat Genet 39: 311–318. 17277777 10.1038/ng1966

[16] He HH, Meyer CA, Shin H, Bailey ST, Wei G, et al (2010) Nucleosome dynamics define transcriptional enhancers. Nat Genet 42: 343–347. 20208536 10.1038/ng.545 PMC2932437

[17] Cuddapah S, Jothi R, Schones DE, Roh TY, Cui K, et al (2009) Global analysis of the insulator binding protein CTCF in chromatin barrier regions reveals demarcation of active and repressive domains. Genome Res 19: 24–32. 19056695 10.1101/gr.082800.108 PMC2612964

[18] Dowell RD (2010) Transcription factor binding variation in the evolution of gene regulation. Trends Genet 26: 468–475. 20864205 10.1016/j.tig.2010.08.005

[19] Park PJ (2009) ChIP-seq: advantages and challenges of a maturing technology. Nat Rev Genet 10: 669–680. 19736561 10.1038/nrg2641 PMC3191340

[20] Kharchenko PV, Tolstorukov MY, Park PJ (2008) Design and analysis of ChIP-seq experiments for DNA-binding proteins. Nat Biotechnol 26: 1351–1359. 19029915 10.1038/nbt.1508 PMC2597701

[21] Ernst J, Plasterer HL, Simon I, Bar-Joseph Z (2010) Integrating multiple evidence sources to predict transcription factor binding in the human genome. Genome Res 20: 526–536. 20219943 10.1101/gr.096305.109 PMC2847756

[22] Won KJ, Ren B, Wang W (2010) Genome-wide prediction of transcription factor binding sites using an integrated model. Genome Biol 11: R7. 20096096 10.1186/gb-2010-11-1-r7 PMC2847719

[23] Pique-Regi R, Degner JF, Pai AA, Gaffney DJ, Gilad Y, et al (2011) Accurate inference of transcription factor binding from DNA sequence and chromatin accessibility data. Genome Res 21: 447–455. 21106904 10.1101/gr.112623.110 PMC3044858

[24] Wang J, Zhuang J, Iyer S, Lin X, Whitfield TW, et al (2012) Sequence features and chromatin structure around the genomic regions bound by 119 human transcription factors. Genome Res 22: 1798–1812. 22955990 10.1101/gr.139105.112 PMC3431495

[25] Bell O, Tiwari VK, Thoma NH, Schubeler D (2011) Determinants and dynamics of genome accessibility. Nat Rev Genet 12: 554–564. 21747402 10.1038/nrg3017

[26] Bannister AJ, Kouzarides T (2011) Regulation of chromatin by histone modifications. Cell Res 21: 381–395. 21321607 10.1038/cr.2011.22 PMC3193420

[27] Zaret KS, Carroll JS (2011) Pioneer transcription factors: establishing competence for gene expression. Genes Dev 25: 2227–2241. 22056668 10.1101/gad.176826.111 PMC3219227

[28] Henikoff S, Shilatifard A (2011) Histone modification: cause or cog? Trends Genet 27: 389–396. 21764166 10.1016/j.tig.2011.06.006

[29] Angelov D, Lenouvel F, Hans F, Muller CW, Bouvet P, et al (2004) The histone octamer is invisible when NF-kappaB binds to the nucleosome. J Biol Chem 279: 42374–42382. 15269206 10.1074/jbc.M407235200

[30] Hoffman BG, Robertson G, Zavaglia B, Beach M, Cullum R, et al (2010) Locus co-occupancy, nucleosome positioning, and H3K4me1 regulate the functionality of FOXA2-, HNF4A-, and PDX1-bound loci in islets and liver. Genome Res 20: 1037–1051. 20551221 10.1101/gr.104356.109 PMC2909568

[31] Zlatanova J, Thakar A (2008) H2A.Z: view from the top. Structure 16: 166–179. 18275809 10.1016/j.str.2007.12.008

[32] Song Q, Smith AD (2011) Identifying dispersed epigenomic domains from ChIP-Seq data. Bioinformatics 27: 870–871. 21325299 10.1093/bioinformatics/btr030 PMC3051331

[33] Barski A, Zhao K (2009) Genomic location analysis by ChIP-Seq. J Cell Biochem 107: 11–18. 19173299 10.1002/jcb.22077 PMC3839059

[34] Boyle AP, Davis S, Shulha HP, Meltzer P, Margulies EH, et al (2008) High-resolution mapping and characterization of open chromatin across the genome. Cell 132: 311–322. 18243105 10.1016/j.cell.2007.12.014 PMC2669738

[35] Li L, He S, Sun JM, Davie JR (2004) Gene regulation by Sp1 and Sp3. Biochem Cell Biol 82: 460–471. 15284899 10.1139/o04-045

[36] Srinivasan L, Atchison ML (2004) YY1 DNA binding and PcG recruitment requires CtBP. Genes Dev 18: 2596–2601. 15520279 10.1101/gad.1228204 PMC525539

[37] Wen B, Wu H, Shinkai Y, Irizarry RA, Feinberg AP (2009) Large histone H3 lysine 9 dimethylated chromatin blocks distinguish differentiated from embryonic stem cells. Nat Genet 41: 246–250. 19151716 10.1038/ng.297 PMC2632725

[38] Hawkins RD, Hon GC, Lee LK, Ngo Q, Lister R, et al (2010) Distinct epigenomic landscapes of pluripotent and lineage-committed human cells. Cell Stem Cell 6: 479–491. 20452322 10.1016/j.stem.2010.03.018 PMC2867844

[39] Zang C, Schones DE, Zeng C, Cui K, Zhao K, et al (2009) A clustering approach for identification of enriched domains from histone modification ChIP-Seq data. Bioinformatics 25: 1952–1958. 19505939 10.1093/bioinformatics/btp340 PMC2732366

[40] Xu H, Wei CL, Lin F, Sung WK (2008) An HMM approach to genome-wide identification of differential histone modification sites from ChIP-seq data. Bioinformatics 24: 2344–2349. 18667444 10.1093/bioinformatics/btn402

[41] Wagner EJ, Carpenter PB (2012) Understanding the language of Lys36 methylation at histone H3. Nat Rev Mol Cell Biol 13: 115–126. 22266761 10.1038/nrm3274 PMC3969746

[42] Strahl BD, Allis CD (2000) The language of covalent histone modifications. Nature 403: 41–45. 10638745 10.1038/47412

[43] Ravasi T, Suzuki H, Cannistraci CV, Katayama S, Bajic VB, et al (2010) An atlas of combinatorial transcriptional regulation in mouse and man. Cell 140: 744–752. 20211142 10.1016/j.cell.2010.01.044 PMC2836267

[44] Quinlan AR, Hall IM (2010) BEDTools: a flexible suite of utilities for comparing genomic features. Bioinformatics 26: 841–842. 20110278 10.1093/bioinformatics/btq033 PMC2832824

[45] Mortazavi A, Williams BA, McCue K, Schaeffer L, Wold B (2008) Mapping and quantifying mammalian transcriptomes by RNA-Seq. Nat Methods 5: 621–628. 18516045 10.1038/nmeth.1226 PMC13303166

[46] Johnson DS, Mortazavi A, Myers RM, Wold B (2007) Genome-wide mapping of in vivo protein-DNA interactions. Science 316: 1497–1502. 17540862 10.1126/science.1141319

[47] Zhang Y, Liu T, Meyer CA, Eeckhoute J, Johnson DS, et al (2008) Model-based analysis of ChIP-Seq (MACS). Genome Biol 9: R137. 18798982 10.1186/gb-2008-9-9-r137 PMC2592715

[48] Nix DA, Courdy SJ, Boucher KM (2008) Empirical methods for controlling false positives and estimating confidence in ChIP-Seq peaks. BMC Bioinformatics 9: 523. 19061503 10.1186/1471-2105-9-523 PMC2628906

[49] Reorganizing the protein space at the Universal Protein Resource (UniProt). Nucleic Acids Res 40: D71–75. 22102590 10.1093/nar/gkr981 PMC3245120

