# Supplementary figures and images for: The Patterns of Histone Modifications in the Vicinity of Transcription Factor Binding Sites in Human Lymphoblastoid Cell Lines
Source: PLoS One. 2013 Mar 19;8(3):e60002. doi: 10.1371/journal.pone.0060002 (PMC3602107; doi:10.1371/journal.pone.0060002)

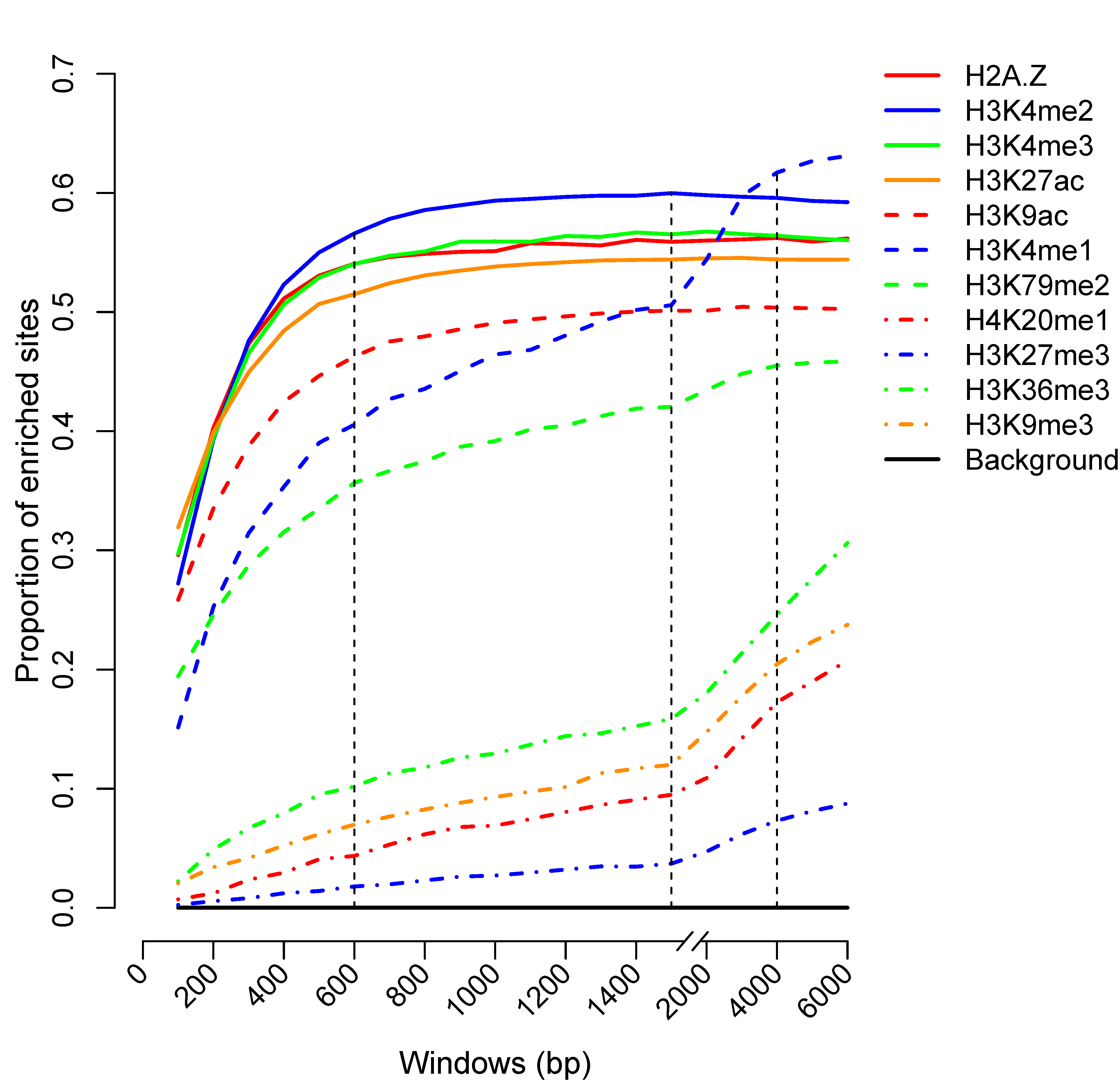

Supplement: Figure S1 — Proportion of ChIP-seq binding sites enriched with histone marks in different windows. (TIF) [file pone.0060002.s001.tif]

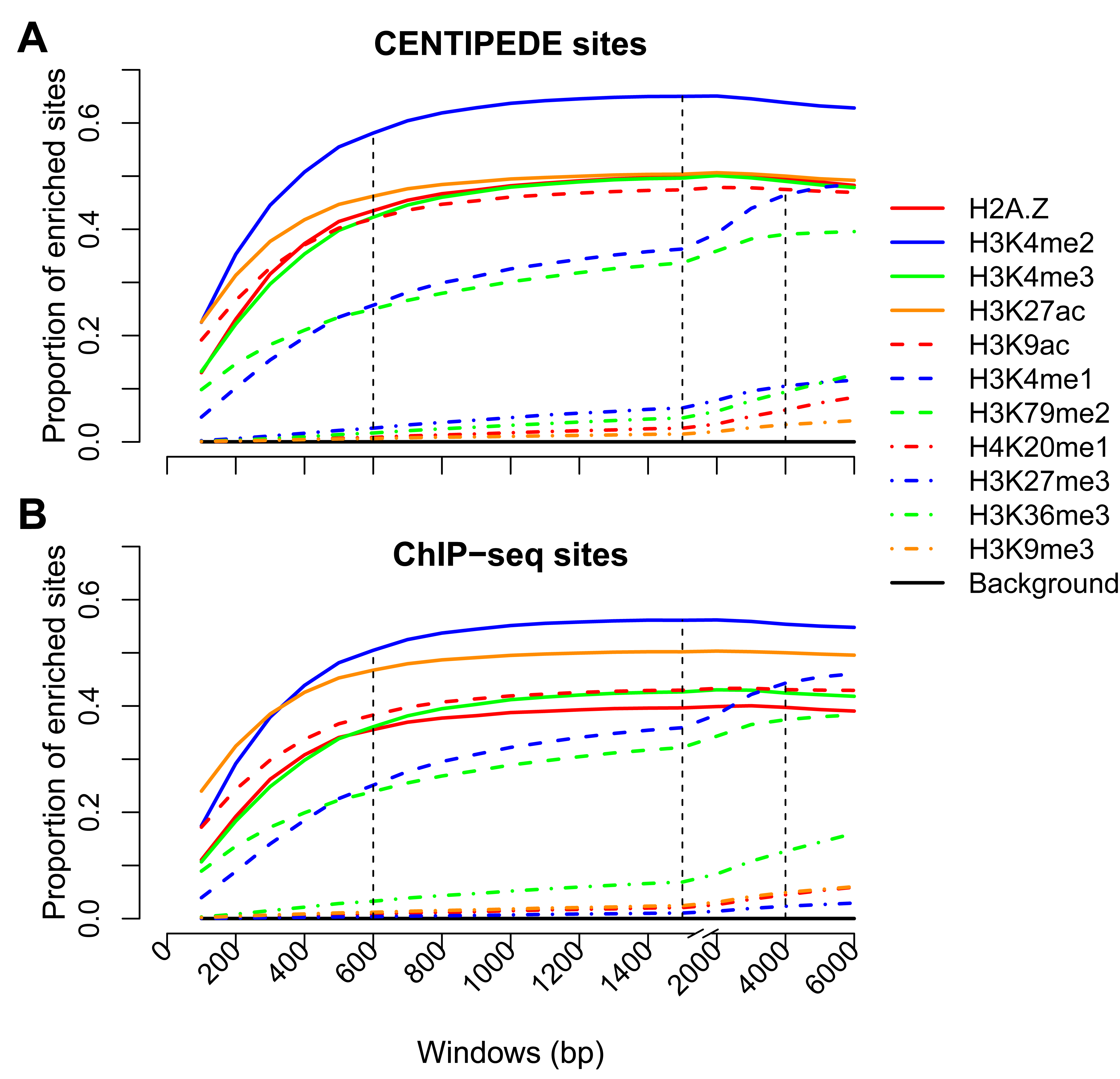

Supplement: Figure S2 — Proportion of enriched CENTIPEDE (A) and ChIP-seq (B) sites when a sampling approach was used. (TIF) [file pone.0060002.s002.tif]

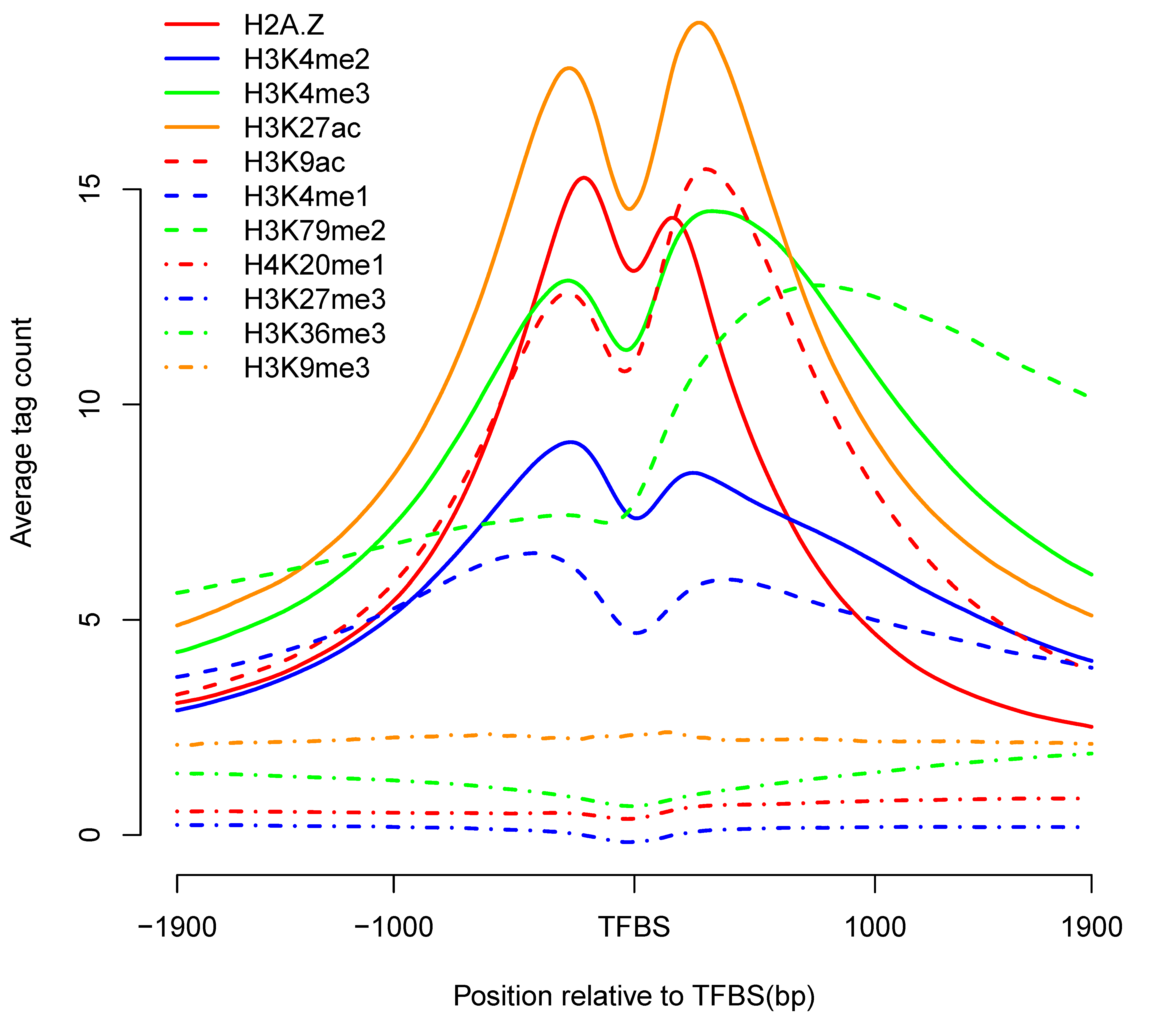

Supplement: Figure S3 — Patterns of 11 histone marks around ChIP-seq binding sites. (TIF) [file pone.0060002.s003.tif]

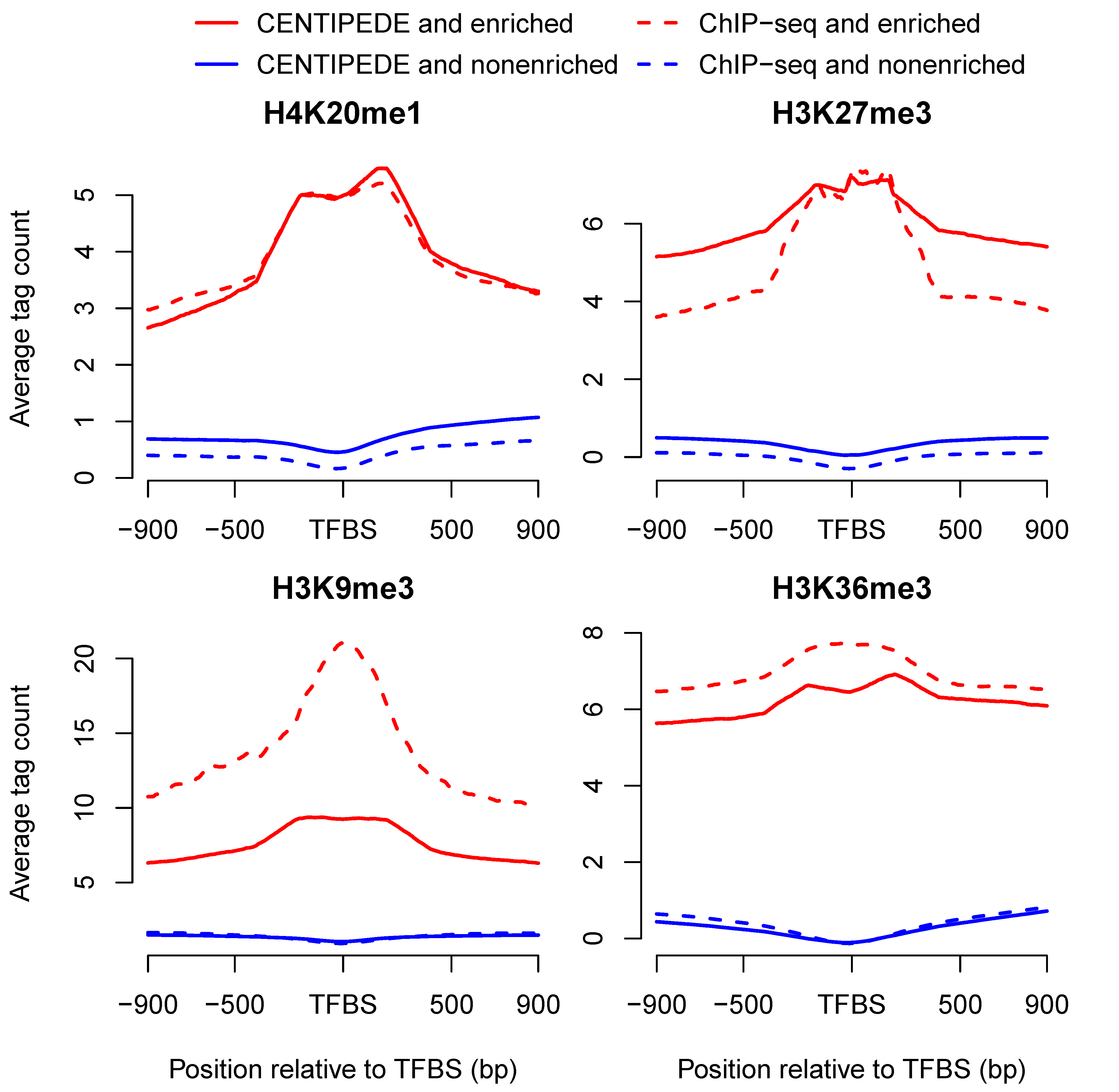

Supplement: Figure S4 — Patterns of long-range marks around CENTIPEDE and ChIP-seq binding sites. (TIF) [file pone.0060002.s004.tif]

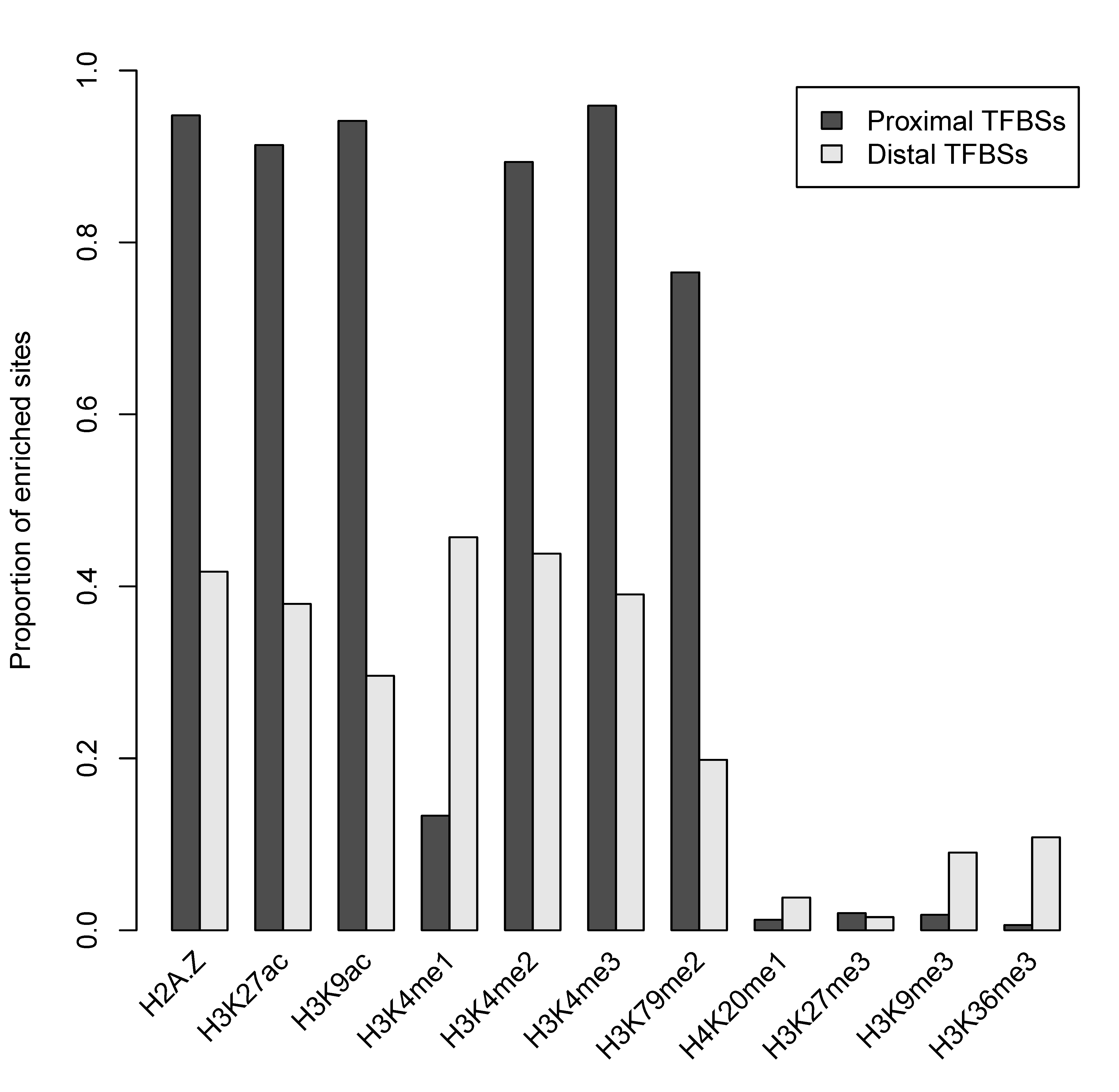

Supplement: Figure S5 — Proportion of enriched proximal and distal sites by ChIP-seq in a 600-bp window. (TIF) [file pone.0060002.s005.tif]

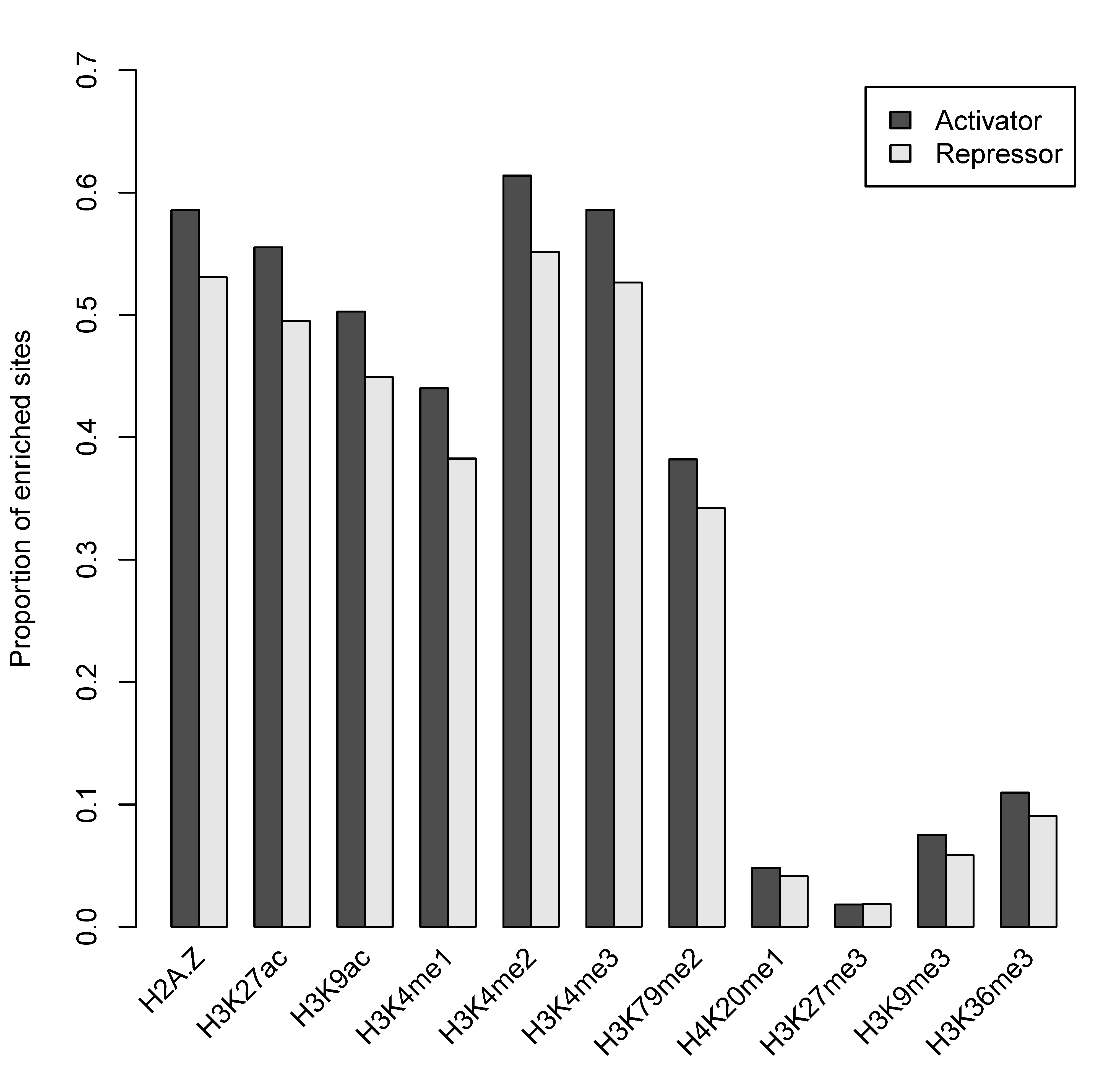

Supplement: Figure S6 — Proportion of enriched activator and repressor sites by ChIP-seq in a 600-bp window. (TIF) [file pone.0060002.s006.tif]

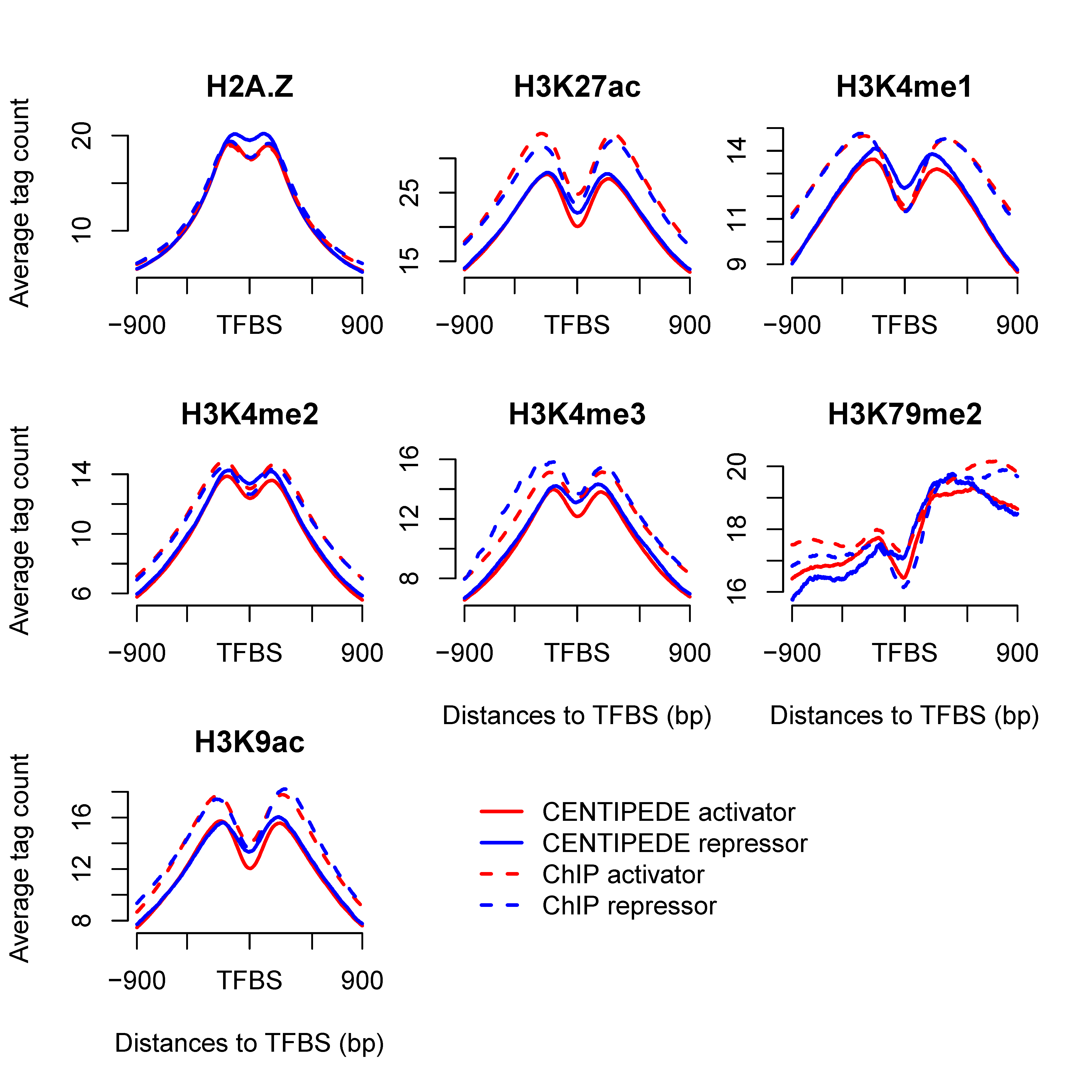

Supplement: Figure S7 — Patterns of seven short-range marks around enriched activator and repressor sites in the distal regions inferred by CENTIPEDE and ChIP-seq. (TIF) [file pone.0060002.s007.tif]

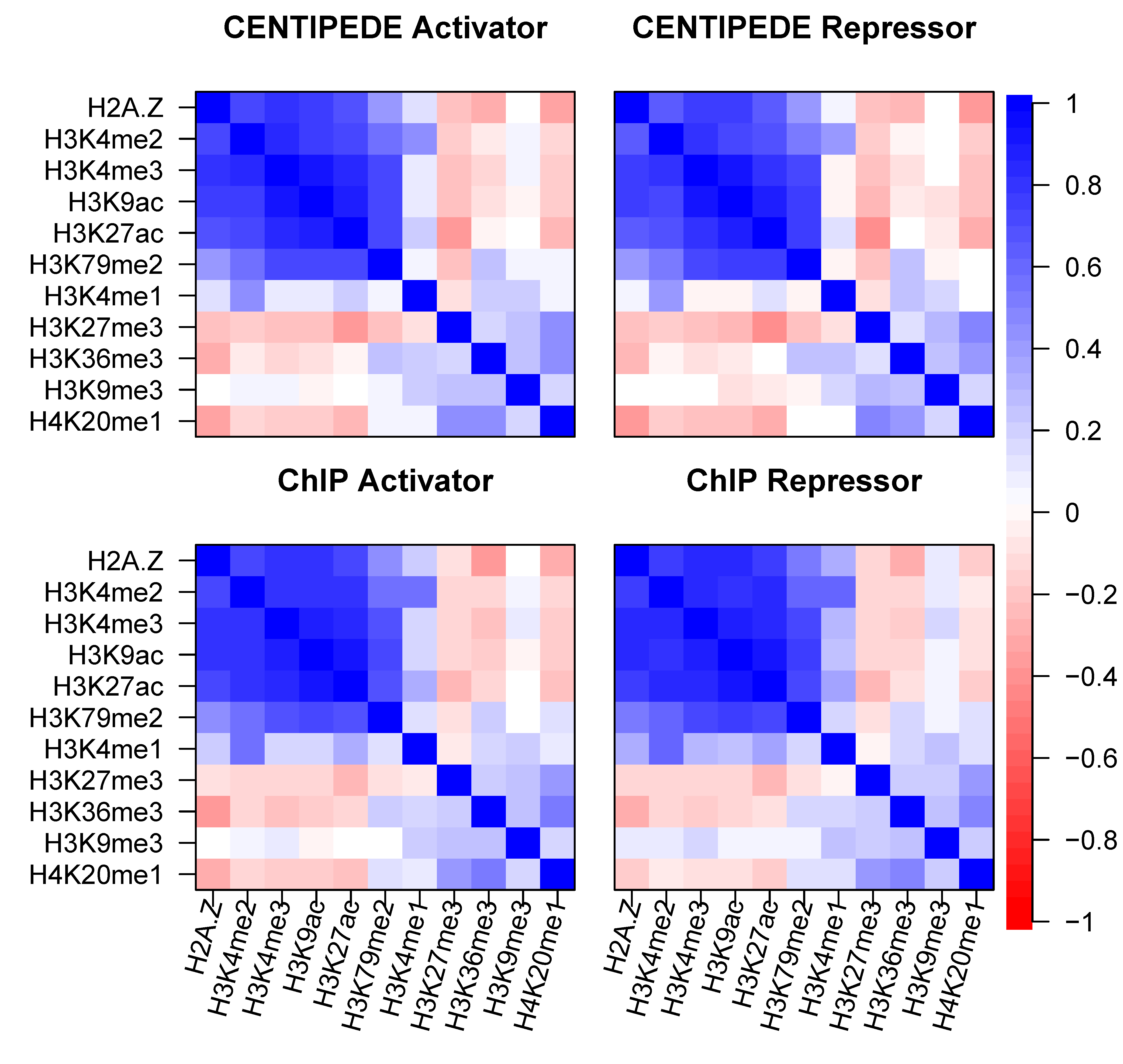

Supplement: Figure S8 — Correlations of histone marks at activator and repressor binding sites. (TIF) [file pone.0060002.s008.tif]

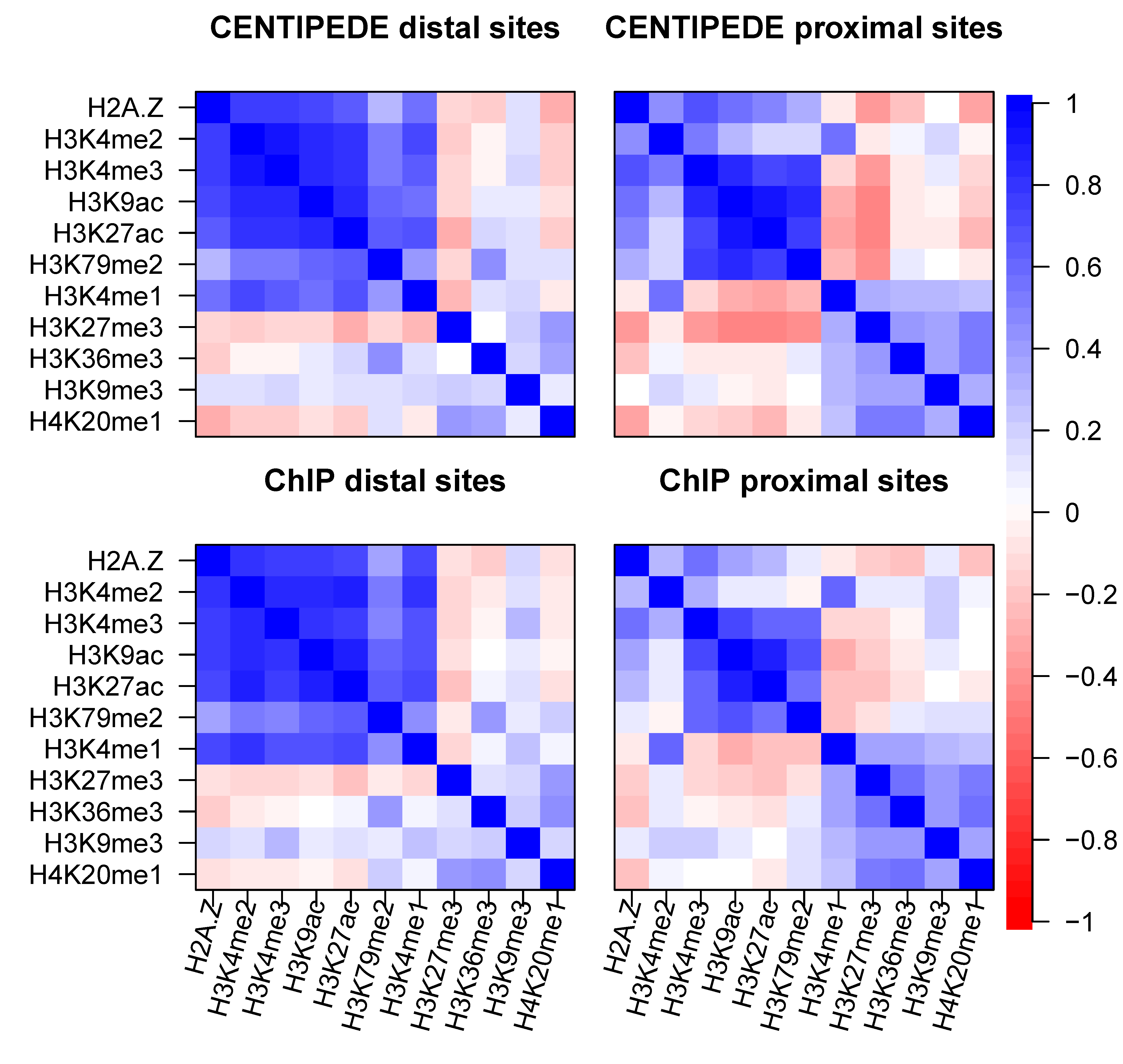

Supplement: Figure S9 — Correlations of histone marks at distal and proximal binding sites. (TIF) [file pone.0060002.s009.tif]
